# Electrospun Chitosan/Collagen Fibers Incorporating PLGA and Hydroxyapatite Nanoparticles for In-Vitro 3T3 Fibroblast Migration

**DOI:** 10.3390/polym18161964

**Published:** 2026-08-11

**Authors:** Laila Procel-Badillo, Sarah Briceño, Lenin Ramírez, Gema González

**Affiliations:** 1 School of Physical Sciences and Nanotechnology, Yachay Tech University, Urcuquí 100119, Ecuador; laila.procel@yachaytech.edu.ec; 2 School of Biological Sciences and Biomedicine, Yachay Tech University, Urcuquí 100119, Ecuador; lramirez@yachaytech.edu.ec

**Keywords:** electrospinning, Chitosan/Collagen composite scaffolds, hydroxyapatite nanoparticles, fibroblast migration

## Abstract

The development of 3D scaffolds that enable the reliable evaluation of cell migration remains a critical challenge in tissue engineering and wound-healing-related research. In this work, 3D scaffolds based on Chitosan/Collagen/Poly(lactic-co-glycolic acid) (PLGA)/Hydroxyapatite (HAp) nanoparticles are presented as a promising approach to studying the migration of the 3T3 cell line. The electrospinning technique was employed to fabricate fibers with an average diameter of 0.2 μm for CH/Coll, 0.69 μm for CH/Coll/PLGA, and 0.14 μm for CH/Coll/HAp. The electrospinning parameters were optimized, and the properties of the scaffolds were further enhanced by incorporating hydroxyapatite (HAp) and PLGA, thereby improving their regenerative potential and tissue-engineering applicability. Preliminary cell proliferation was monitored in the wound area at 4, 12, 24, and 35 h. The highest cell migration was observed in scaffolds with hydroxyapatite nanoparticles. The resulting fiber matrix demonstrated promising effects on fibroblast migration in an in vitro scratch-assay model using NIH 3T3 cells, relevant to skin tissue repair.

## 1. Introduction

The restoration of damaged skin remains a major challenge in regenerative medicine due to the complexity of the wound-healing process, which involves inflammation and proliferation. These processes rely on coordinated cell migration, extracellular matrix (ECM) deposition, and biochemical signalling to restore tissue integrity [1]. The extracellular matrix plays a central role by providing both structural support and biochemical cues that regulate fibroblast adhesion, migration, and differentiation [2]. Consequently, the development of 3D scaffolds capable of replicating the extracellular matrix has become a key strategy in designing advanced wound-healing platforms.

Among these strategies, electrospun fibrous scaffolds have attracted significant attention because their nanoscale and microscale architectures closely resemble the native extracellular matrix. Fibers obtained by electrospinning exhibit high surface area and porosity, enabling surface functionalization for specific biomedical applications [3,4]. The electrospinning method enables modulation of fiber physical properties by adjusting process parameters [5]. These optimized characteristics are crucial for designing advanced wound dressings that can mimic the native tissue environment and promote cell migration [6,7].

Despite these advantages, single-component polymeric scaffolds often have important limitations. To address these limitations, nanocomposite-based scaffolds combining natural and synthetic materials with nanoparticles have emerged as a promising approach. Natural polymers such as Chitosan are biocompatible, biodegradable, and antimicrobial, and are structurally similar to glycosaminoglycans, which are a major component of the extracellular matrix (ECM) [8]. Meanwhile, Collagen is one of the most abundant components of the skin’s ECM, representing approximately 70–80% of the dermis’s dry weight. Chitosan/Collagen composite is a potential candidate for use as a 3D scaffold to mimic the skin ECM, which provides structural support and facilitates key cellular functions, including growth, differentiation, and motility, providing complementary performance and synergy, promoting cellular growth, adhesion, proliferation, and cell migration [9,10,11].

Incorporating hydroxyapatite (HAp) and poly(lactic-co-glycolic acid) (PLGA) into Chitosan/Collagen electrospinning solution can significantly enhance their functionality and broaden their applicability in tissue engineering, particularly for skin and bone regeneration [12,13]. Hydroxyapatite nanoparticles (HAp) have been extensively studied for applications in tissue engineering and regenerative medicine [14]. Moreover, its bioactive surface enhances attachment of osteoblasts and fibroblasts, and some studies have reported mild antimicrobial effects due to ionic interactions with bacterial membranes [15]. Although numerous studies have explored the use of PLGA and HAp in Chitosan/Collagen-based scaffolds and membranes, research on their combined integration into electrospun fibers, especially at the microscale, remains limited. PLGA is a biodegradable and biocompatible copolymer widely employed in medical and pharmaceutical applications [16]. Significant fibroblast cell proliferation has been observed with the nanofibrous PLGA/collagen scaffold [17].

Previous studies have extensively investigated Chitosan/Collagen electrospun scaffolds for migration assessment applications [9,18], demonstrating their biocompatibility, extracellular matrix–like architecture, and ability to support fibroblast adhesion and migration. In this work, fibroblast migration on fibers in an artificial wound model was used to observe 3T3 migration, which is required for wound closure. NIH 3T3 cells are a hypertriploid cell line derived from mouse embryonic tissue and are widely used in biological research [19]. The use of 3T3 fibroblast cells with fibers in the migration assessment assay provides essential insights into how fibrous materials to modulate cellular processes.

The novelty of this work lies in integrating chitosan, collagen, hydroxyapatite nanoparticles, and PLGA into a single electrospun fiber scaffold to evaluate fibroblast migration as a function of scaffold composition. Incorporating HAp or PLGA into Chitosan/Collagen electrospun fibers modifies fiber morphology and cell–material interactions, thereby influencing NIH 3T3 fibroblast migration in a scratch-assay model. Unlike previous Chitosan/Collagen [9], Chitosan/HAp [20], or PLGA/Collagen [21] systems, this study demonstrates the additive effect of organic, inorganic, and synthetic components on cell migration, providing preliminary experimental evidence that hydroxyapatite nanoparticle incorporation enhances fibroblast motility within a fibrous matrix.

## 2. Materials and Methods

### 2.1. Materials

Low-molecular-weight chitosan, a partially deacetylated derivative of chitin composed of β-(1-4)-linked D-glucosamine and N-acetyl-D-glucosamine units, with a degree of deacetylation of ≥75%, molecular weight of 50–190 kDa, and viscosity of 20–300 cP, was purchased from Sigma-Aldrich, St. Louis, MO, USA. Hydrolyzed powder collagen (20% viscosity; PEPTAN B 2000 LD Type). Acetic acid (AA) was purchased from Sigma-Aldrich. SprayBase Electrospinning Starter Kit. Trifluoroacetic acid (TFA) was purchased from Sigma-Aldrich. Poly(lactic-co-glycolic acid) (PLGA, 85/15) was purchased from Sigma-Aldrich. Hydroxyapatite (HAp) was synthesized previously following [22,23].

Three solvent systems based on acetic acid (AA), trifluoroacetic acid (TFA), and dichloromethane (DCM) were initially evaluated. TFA was selected as the principal solvent because it provided adequate dissolution of chitosan, collagen, and PLGA and generated more continuous fibers with fewer morphological defects than the acetic-acid formulations evaluated. Chitosan was gradually added to 99% TFA in 5–10 min intervals at room temperature under magnetic stirring at 500 rpm, maintaining an acidic pH. After the addition was complete, the covered solution was stirred continuously for 10–15 h until visually homogeneous. Collagen was gradually added to 99% TFA and stirred at 500 rpm for 1–2 h. The separately prepared CH and collagen solutions were then combined at the required volume ratio and homogenised under continuous magnetic stirring. PLGA was dissolved separately in TFA at 500 rpm for 1–2 h before being added to the CH/Coll blend. HAp was dispersed in TFA in an ultrasonic bath for 20 min, then homogenised, yielding a total dispersion time of approximately 30 min. LMW chitosan, collagen, and PLGA were dissolved separately in 99% TFA at 10 °C with gradual addition and stirring for 10, 2, and 1 h, respectively, minimizing solvent evaporation. HAp was dispersed in 99% TFA using an ultrasonic bath for 20 min and a homogenizer. Polymeric solutions were electrospun to produce fibers. Chitosan and collagen were used as the primary components, with PLGA and HAp incorporated to form composite blends.

### 2.2. Electrospinning

**Figure 1:**
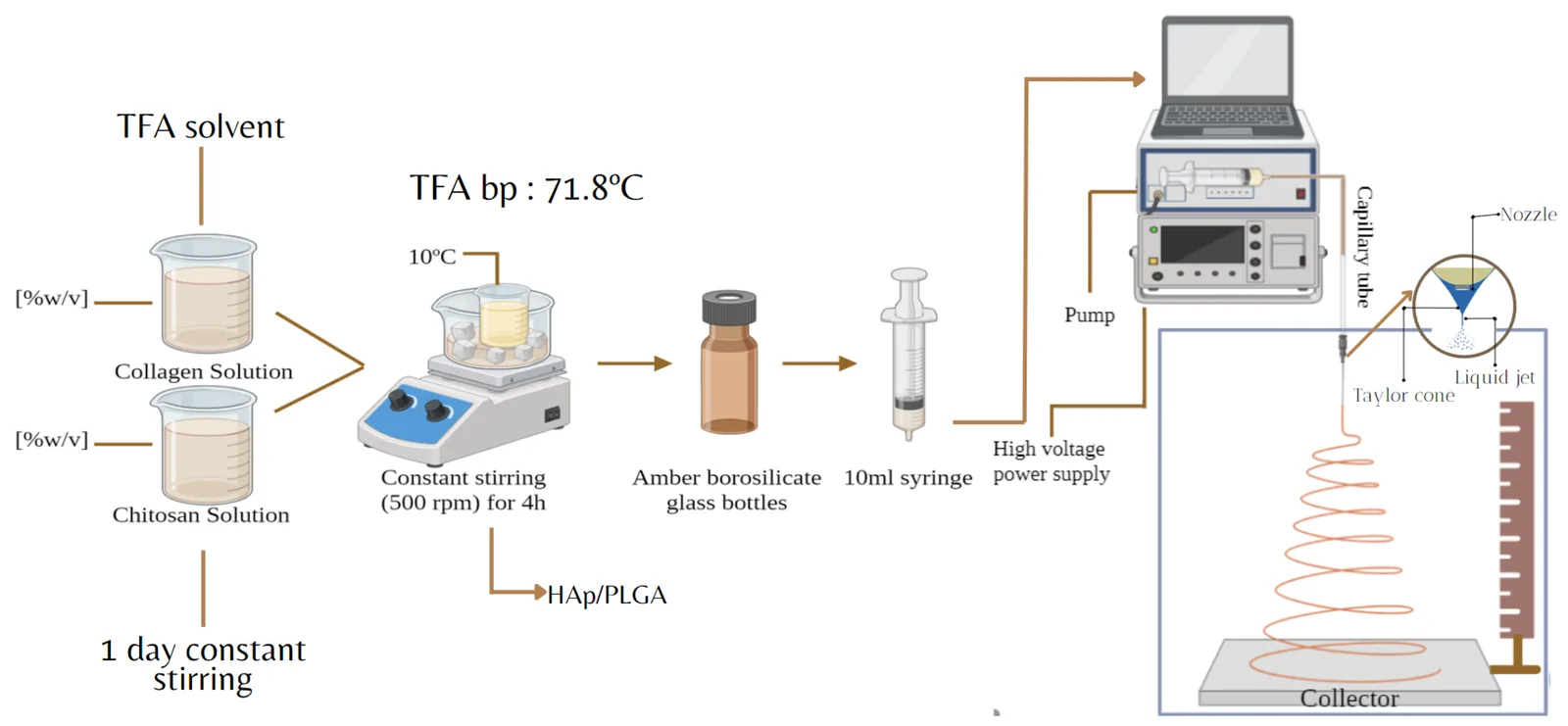
Experimental methodology for preparing Chitosan/Collagen/HAp/PLGA fibers by electrospinning.

The electrospinning equipment to obtain the fibers was the SprayBase^*TM*^ (Figure 1). The fibers were deposited onto aluminum foil, which served as the collector. A DC voltage supply was used to release the polymer solutions at a controlled flow rate between 0.010 mL/h and 0.500 mL/h. An electrical potential of 13–20 kV was applied to the solutions to evaporate the solvents. The distance between the emitter and collector was 12–13 cm. The electrospinning process was performed at room temperature. The specific parameters used for each solution, as presented in Table 1.

### 2.3. 3T3 Fibroblast Cells Migration Assay

#### 2.3.1. Cell Line

The murine cell line NIH/3T3 (Mus musculus) passage 37, which is widely used as an in vitro model of fibroblasts instudies concerning fibroblast migration, cell viability, and wound healing–related processes, was provided by Prof.Fabian Aguilar from the Institute of Histology and Embryology “Dr. Mario G. Burgos” (IHEM), Faculty of MedicalSciences, Universidad Nacional de Cuyo, Mendoza, Argentina [24].

#### 2.3.2. Cell Migration Assay

**Figure 2:**
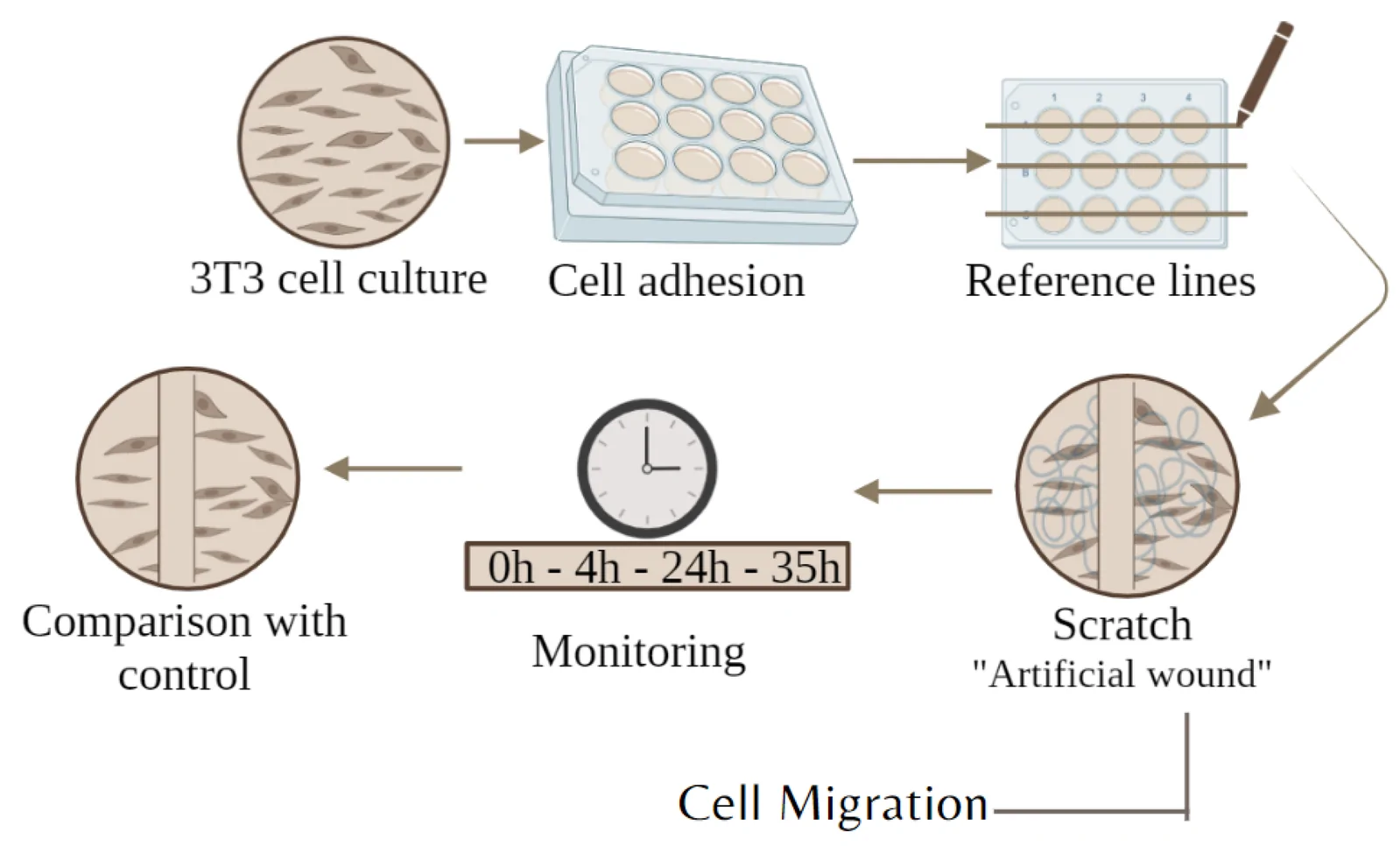
Experimental workflow of the migration assessment assay using 3T3 fibroblast cells. After cell adhesion and scratch formation, wound closure was monitored at 0, 4, 24, and 35 h to evaluate cell migration.

In a sterile environment, 3T3 fibroblasts were cultured in a suitable growth medium, DMEM/F12 supplemented with an antibiotic mix. The DMEM/F12 was supplemented with 3% (positive control), and 10%(for cell growth or negative control) Fetal Bovine Serum (FBS). 1.5 mL was placed in each well, which already contained the fiber samples and controls. Five cell culture plates were seeded for each sample. All culture plates were incubated until reaching 85–95 % confluency. The culture medium was replaced with fresh medium, and the cells were incubated and monitored at 4, 12, 24, and 35 h for wound closure (Figure 2). Before cell seeding, the scaffold samples were exposed to UV light in a sterile environment and then transferred to the culture wells using aseptic techniques. A 1.5 mL aliquot of the homogenized NIH/3T3 cell suspension in DMEM was added to each well containing either a scaffold sample or its corresponding control. Five culture plates were prepared for each formulation, and the cultures were incubated until 90–100% confluence before scratch formation. A cell count was performed using ImageJ, version 1.54t, and a manual count was also performed. Both results were similar; therefore, the average digital count was taken.

## 3. Results

### 3.1. Electrospinning Parameters Optimization

The electrospinning process and fiber formation are influenced by several parameters that determine the final properties of the fiber film. These factors can be categorized into three groups: inherent solution properties (such as polymer concentration/viscosity), solvent system and conductivity, and processing conditions (including voltage, distance, flow rate, and nozzle characteristics) [25]. Together, these parameters optimize the diameter and morphology of the electrospun fiber mesh, which are essential for its applications in wound dressings and tissue engineering scaffolds. Thus, careful optimization of the working parameters and electrospinning variables is necessary to achieve structures with the desired physical and biological performance.

Chitosan at low molecular weight (LMW CH) was selected for its homogeneous dissolution at high concentrations, which enables continuous, stable flow during the electrospinning process. In contrast, high molecular weight chitosan (HMW CH) tends to form highly viscous solutions that frequently cause needle clogging and jet instability during fiber formation, with higher irregularities in fibers [26,27]. LMW chitosan exhibits better dispersion and miscibility, promoting stronger intermolecular interactions, such as hydrogen bonding and ionic interactions, with other polymers, such as poly(lactic-co-glycolic acid) (PLGA), thereby improving fiber uniformity.

After an extensive evaluation of solvents for fiber preparation, including acetic acid (AA) and dichloromethane (DCM), TFA was selected as the optimal solvent. TFA has a substantially lower pKa than acetic acid. Its stronger acidity promotes protonation of the chitosan amino groups and disrupts intermolecular associations, thereby facilitating dissolution and electrospinning [28]. TFA fluorine atoms exert an electron-withdrawing effect, further weakening the oxygen-hydrogen bond in the acid group and facilitating proton (H+) release. Thus, TFA was selected as the main solvent, as after several attempts with the AA solvent, the fibers remained poorly structured. Fibers with DCM co-solvent were produced because it improves solution conductivity for electrospinnability [29].

**Figure 3:**
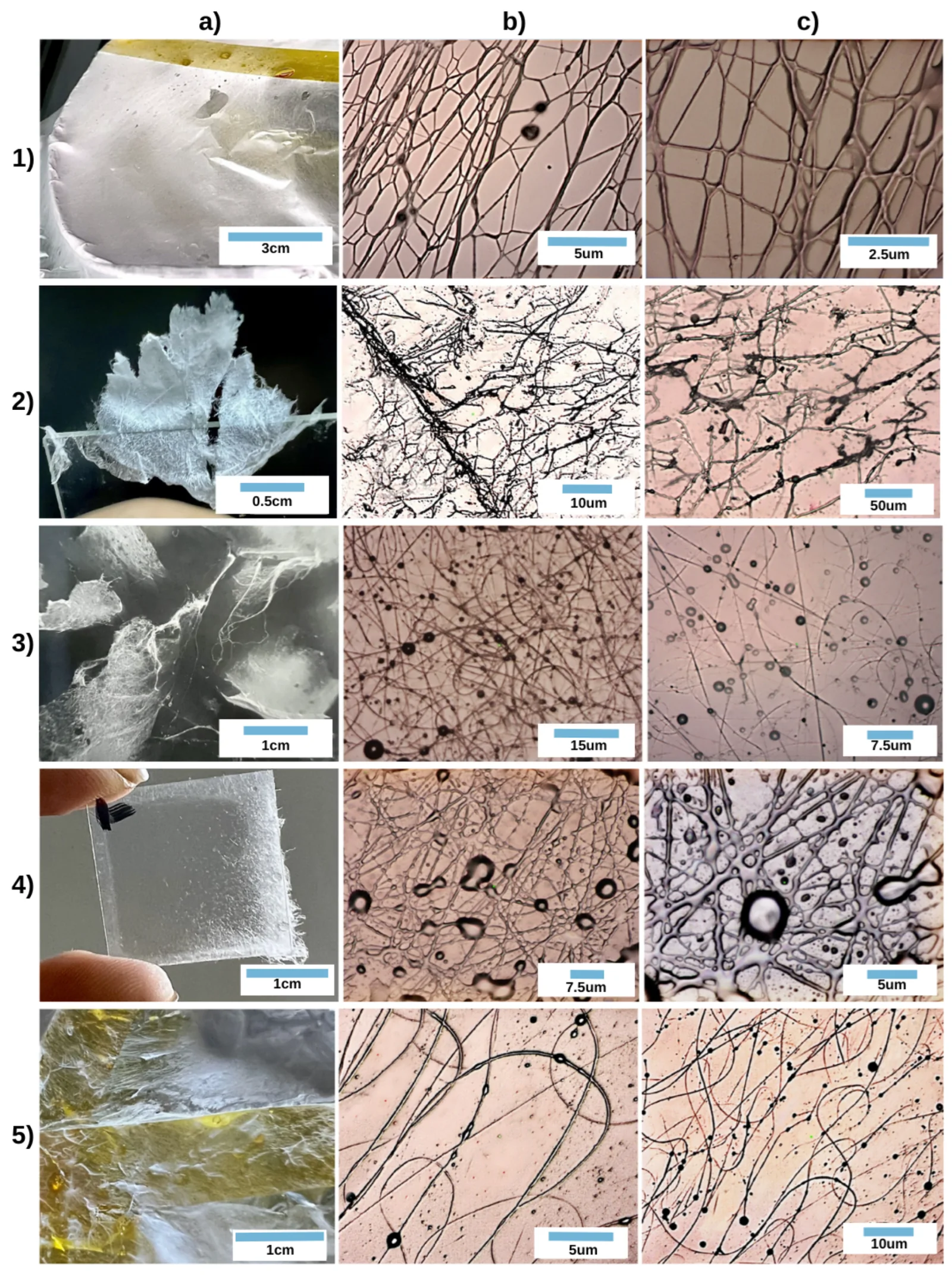
Fibers at three different magnifications: (**1**) CH:Coll, (**2**) Chitosan, (**3**) Collagen, (**4**) CH/Coll/ PLGA, (**5**) CH/Coll/HAp.

The optimized fibers were obtained using chitosan at concentrations of 7% to 10%, dissolved in TFA. All fibers were produced at high voltages (See Figure 3). Voltage directly influences and induces the formation of the Taylor cone. Voltage and solution viscosity are directly correlated with the solution’s physical properties and the efficiency of the electrospinning mechanism.

**Figure 4:**
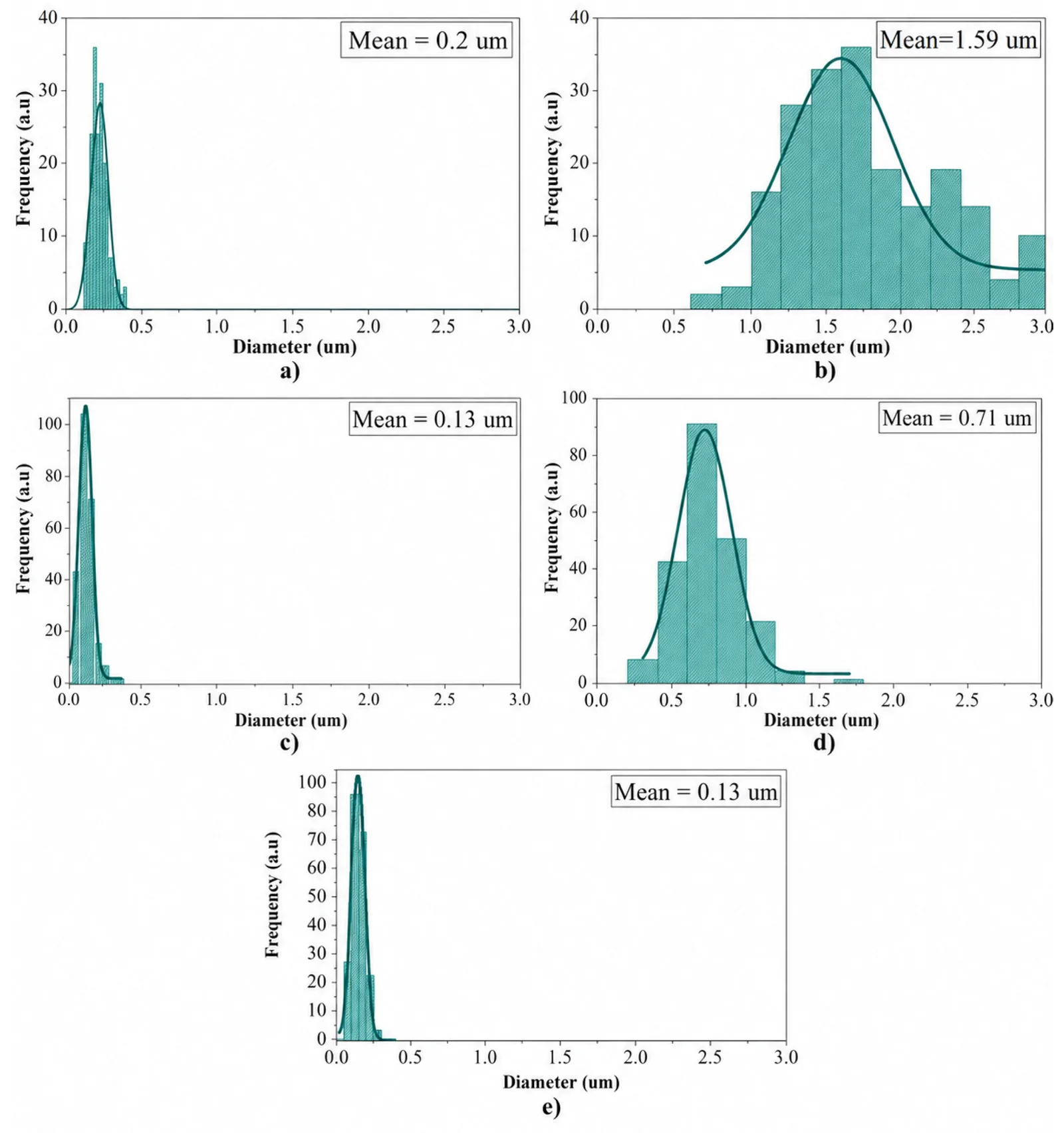
Average diameter of fibers: (**a**) Chitosan/Collagen, (**b**) Chitosan, (**c**) Collagen, (**d**) CH/Coll/ PLGA, (**e**) CH/Coll/HAp.

The choice of an appropriate needle is essential in the electrospinning process; the needle’s internal diameter determines the size of the polymer solution droplet that can be electrospun, directly affecting the uniformity and fiber diameter. Needles with smaller internal diameters favor the formation of finer fibers because less solution is processed, resulting in a thinner electrospinning jet. The geometry of the needle tip can influence the electric field in the droplet region, thereby modifying the jet’s stability and, consequently, the quality of the fibers. During electrospinning, the viscosity of the polymer solution plays a crucial role in determining the jet stability and the resulting fiber morphology. As the solution viscosity increases, the polymer chains become more entangled, requiring a stronger electric field to overcome the surface tension and initiate ejection from the Taylor cone. Consequently, a higher applied voltage is required to fabricate continuous, uniform fibers from more viscous solutions. Therefore, fibers obtained with a higher needle require higher voltages and tend to have higher average diameters (See Figure 4). The finer fibers are produced with collagen (See Figure 4), while chitosan fibers were produced with 1.2–1.6 mm, which are coarser. The collagen solution was less viscous; thus, a smaller-diameter needle (0.55 mm) was used, and voltages of 14–17 kV were required.

The most well-defined and continuous fibers were obtained from the Chitosan/Collagen (1:1) blend, which was electrospun using a large needle diameter (1.2–1.6 mm), requiring a correspondingly higher applied voltage. The incorporation of 1 wt% hydroxyapatite (HAp) into the CH/Coll solution led to a reduction in viscosity, as HAp was previously dispersed in trifluoroacetic acid (TFA), thereby increasing the overall TFA proportion in the mixture. The resulting lower viscosity facilitated the formation of fibers with smaller average diameters (See Figure 4). In contrast, the addition of PLGA to the CH/Coll solution increased viscosity due to its high molecular weight and flexible aliphatic chains. PLGA and the biopolymers coexist as a partially miscible system, in which weak intermolecular interactions (hydrogen bonding and dipole–dipole forces) form a semistable network that enhances fiber uniformity. Electrospinning of this latter formulation was performed using a medium needle (0.7 mm) at an applied voltage of 14–16 kV, yielding thicker fibers compared to all other blends, except for pure chitosan (See Figure 4).

### 3.2. Optical Microscopy

A 10-cm-diameter film of fibers was produced in 15 min and collected on a Petri dish. In Figure 4. In Figure 3(1a–1c) chitosan dissolved in TFA:DCM, 1:1 at 18 kV as is established in Table 1. This film features continuous, well-structured fibers, with a low bead content and an average diameter of 0.2 μm. This film exhibits a random distribution of fibers and superior uniformity compared to other concentration membranes. Pure chitosan fibers (Figure 3(2a–2c)) exhibited the broadest diameter distribution, which can be attributed to the combined effects of the high viscosity and polyelectrolyte character. Strong chitosan hydrogen bonding, uneven chain entanglement, and extensive protonation of the amino groups in TFA may generate fluctuations in charge density, Taylor-cone stability, jet stretching, and solvent evaporation. Fibers 3.3, 3.4, and 3.5are randomly distributed in a dense network that creates a mesh-like structure, with an average diameter of 1.59 μm, 0.13 μm, 0.71 μm, and 0.14 μm, respectively. These membranes exhibit beads, which could be reduced by adjusting solution composition or electrospinning parameters, or by using electrospinning equipment capable of higher voltages. The occurrence of beads on these fibers can be attributed to irregularities in the electric field, jet instabilities, and inadequate solution viscosity/conductivity, all of which contribute to bead formation.

Figure 4 contains histograms of the average diameters of fibers 1, 2, 3, 4, and 5. The smaller diameter of fibers was produced by the collagen solution, in which a 0.55 mm needle size was used, and the larger diameter of fibers was produced with CH/Coll/PLGA solutions, in which a 0.7 mm needle size was used. The internal diameter of the needle strongly influences fiber morphology in electrospinning; larger needles typically produce thicker fibers due to higher flow rates, while smaller needles favour finer and more uniform fibers, though with increased risk of clogging [30].

### 3.3. Raman Spectroscopy and Thermogravimetric Analysis

**Figure 5:**
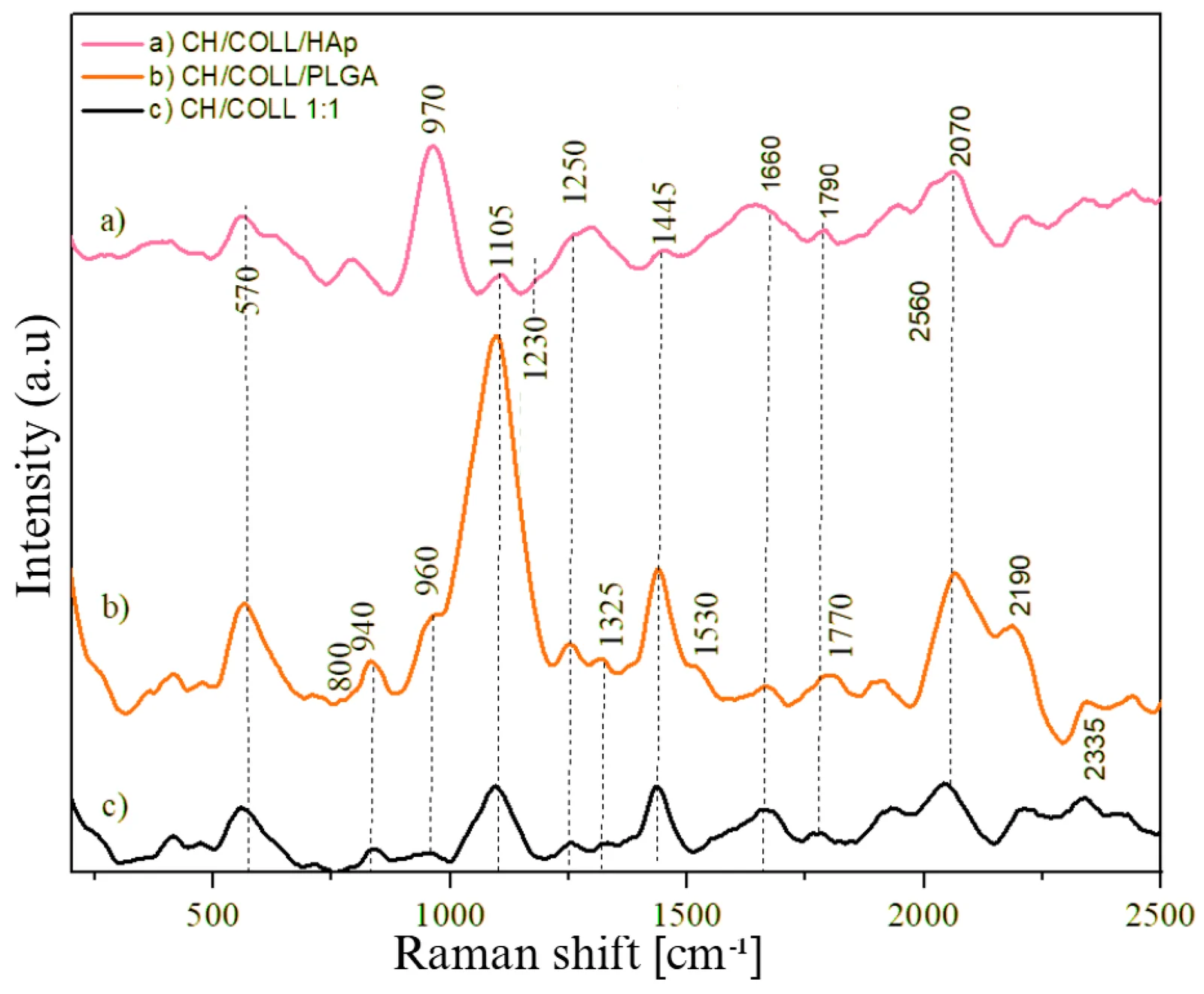
Raman spectra of (a) CH/Coll/HAp, (b) CH/Coll/PLGA, and (c) CH:Coll.

The molecular structure of chitosan and collagen and their interaction is shown in Figure 5. In all (a) CH/Coll/HAp, (b) CH/Coll/PLGA, and (c) CH:Coll, it is observed at 1445 cm^−1^, a band attributed to the CH_2_ vibrations, reflecting the presence of the acetylated or deacetylated units in the chitosan. The bands at 1105 cm^−1^ represent the vibrations of C-O-C bonds characteristic of the glycoside linkages. The band at 1325 cm^−1^ corresponds to CH_2_ vibrations. Collagen presence is evident in all three samples, with amide peaks well defined in 1250 cm^−1^ corresponding to the Amide III and 1660 cm^−1^ corresponding to the Amide I. CH/COLL fibers with PLGA and HAP added present representative peaks, in (a) it is notable the presence of phosphate group at 970 cm^−1^ [31,32], in (b) The peaks at 1325–1530 cm^−1^ associated with out-of-plane deformation vibrations of C-H bonds and with vibrations, indicating the presence of N-H and O-H functional groups, also in (b) a vibration of the carbonyl group (C=O) appears, which is very characteristic and prominent of PLGA at 1770 cm^−1^. Due to the low concentrations of hydroxyapatite and PLGA, other well-defined intensity peaks are not clearly observable.

**Figure 6:**
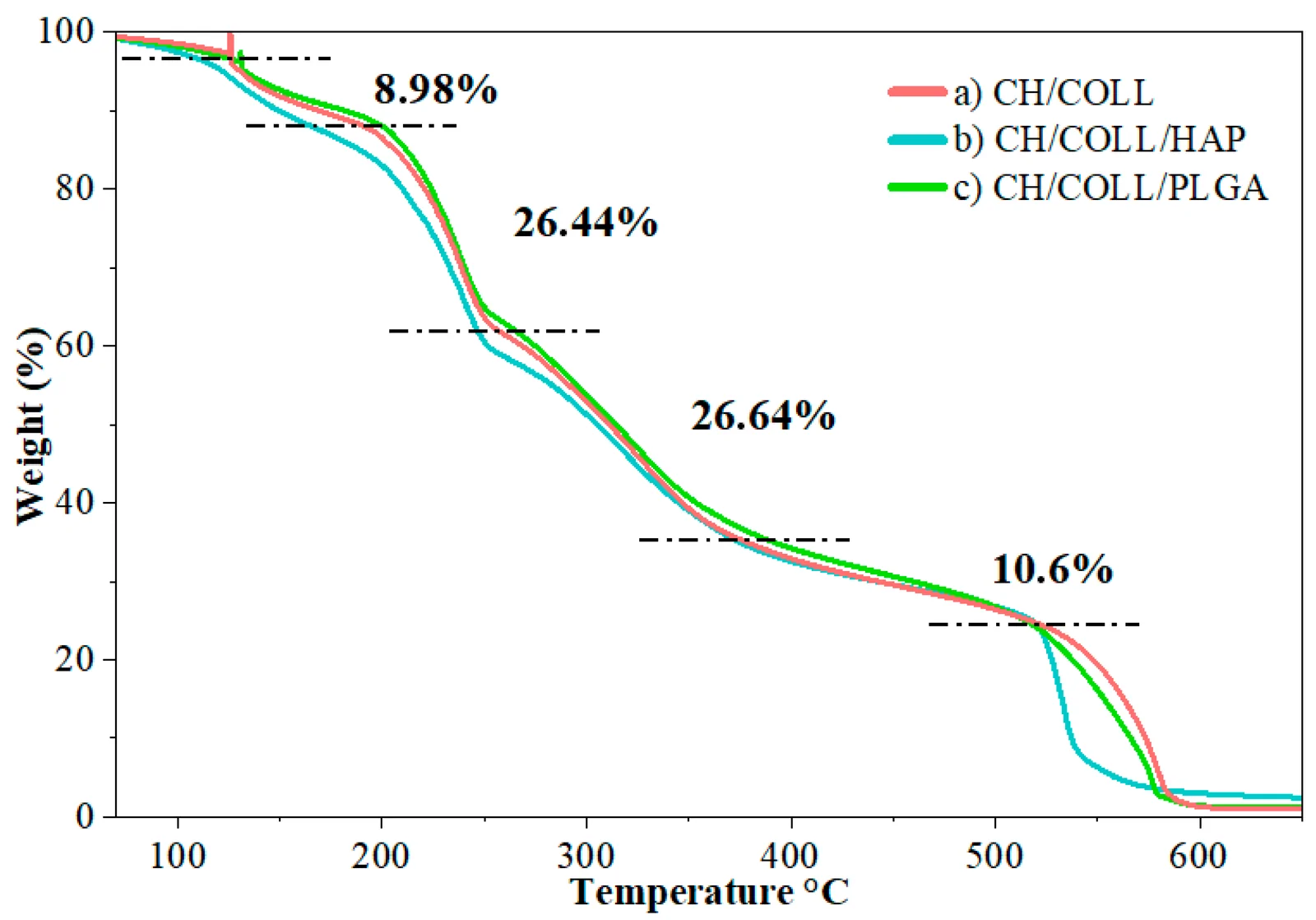
Thermogravimetric analysis of (a) CH/Coll, (b) CH/Coll/HAp, and (c) CH/Coll/PLGA.

TGA of Chitosan/Collagen fibers provides information on the thermal stability of the membranes. In Figure 6 and Table 2, the initial weight loss is attributed to the evaporation of water physically adsorbed on the chitosan surface and residual solvents [33]. The first decomposition of 8.98% is due to the dehydration of volatile functional groups. At this stage, collagen begins to denature (triple-helix thermocollapse), a process that generally occurs at lower temperatures than those at which chitosan denatures [34]. Consequently, a 26.44% weight loss occurs; chitosan undergoes partial deacetylation [34] with significant decomposition of both chitosan and collagen, during which the polymer bonds break [35]. The next stage shows a weight loss of 26.64%, where the initial mass is completely lost due to the decomposition of collagen and chitosan. From 300 °C to 400 °C, the primary thermal decomposition of PLGA occurs. The following loss of 10.6% indicates that almost all of the volatile mass has been removed, impurities adhered to the fibers are eliminated, and only minimal residues and impurities remain [36,37]. The remaining residual mass may be associated with the high thermal stability of hydroxyapatite and residual inorganic components, which decompose above 700 °C, forming other calcium phosphate phases. It is shown that the compound’s stability, as measured by its mass loss percentage, remains constant up to 150–200 ºC, indicating that it will not decompose during sterilization processes.

### 3.4. Cell Migration Assay

**Figure 7:**
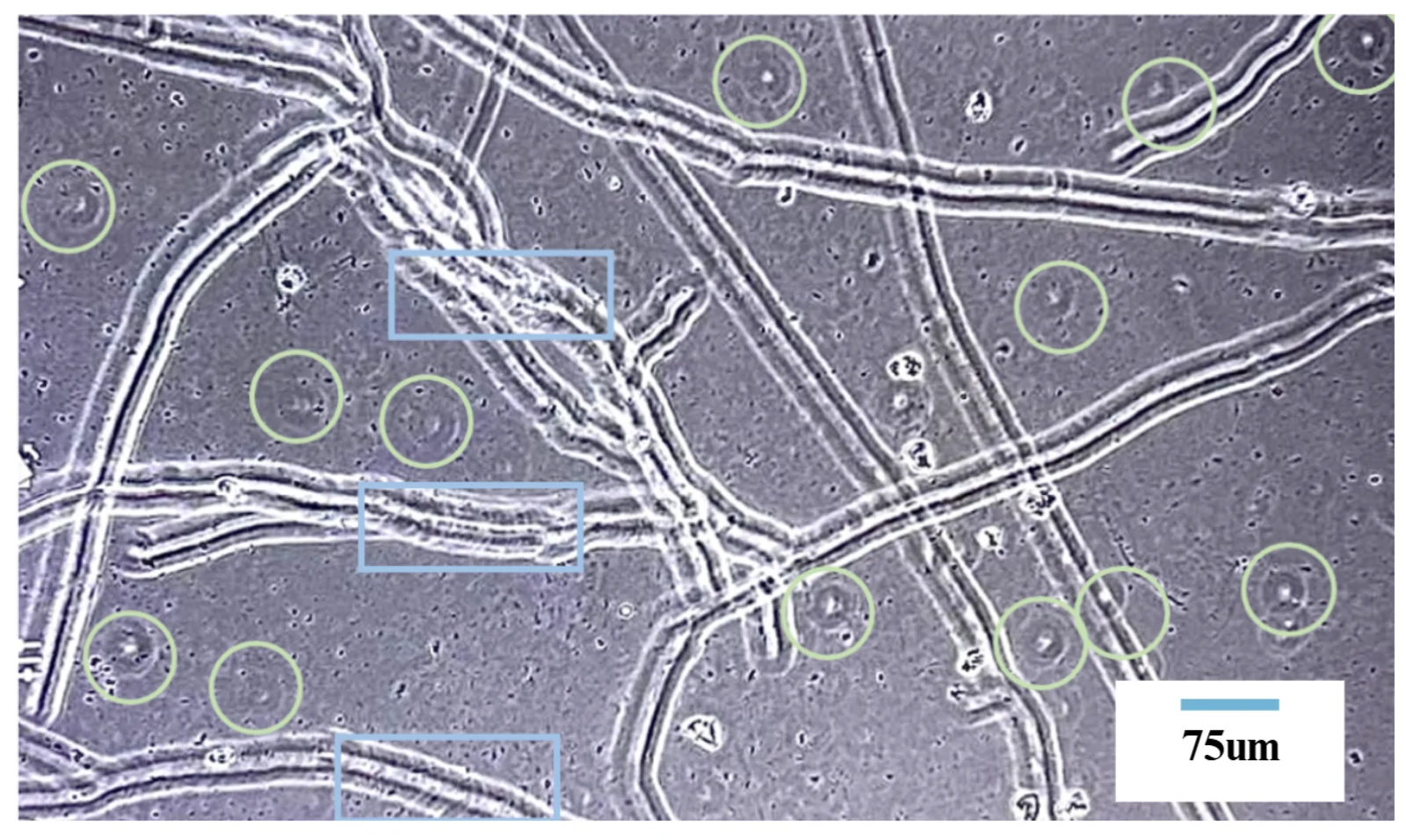
HIT 3T3 fibroblasts adhesion in fibers.

**Figure 8:**
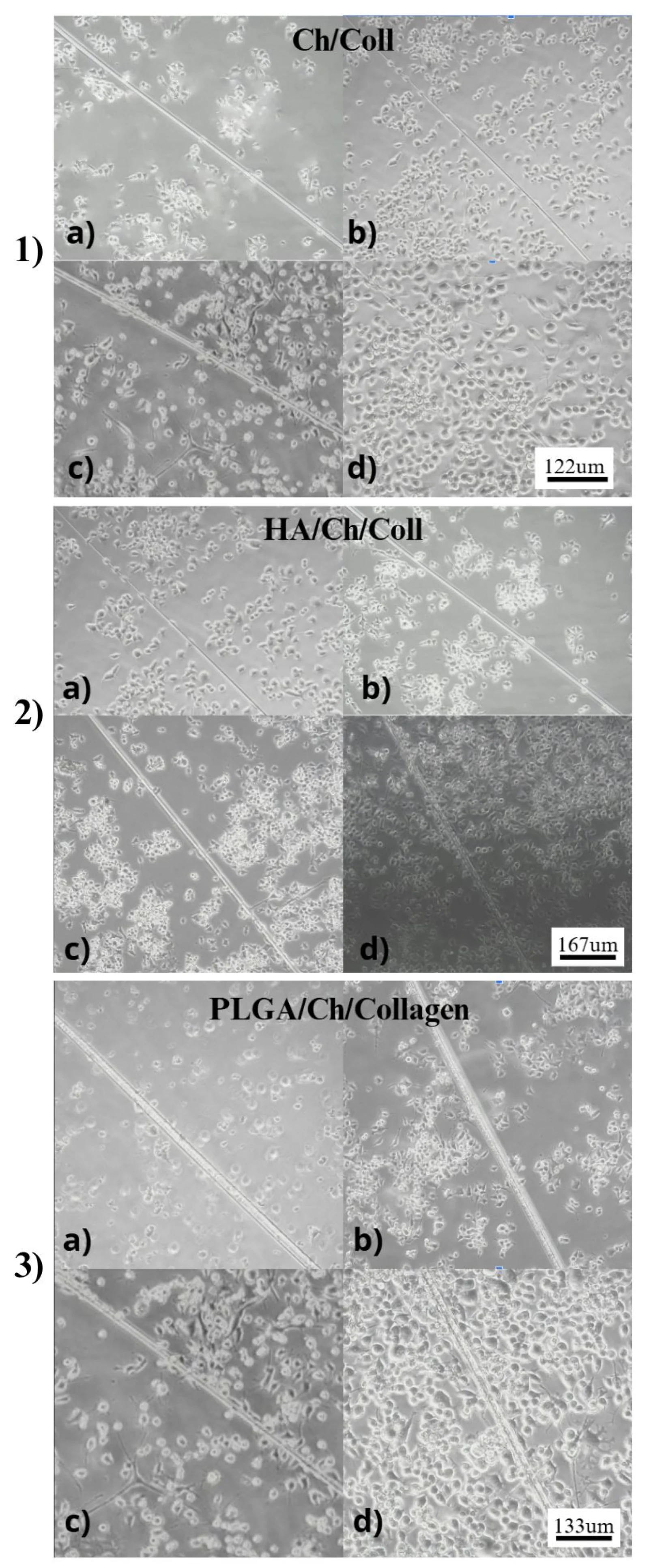
3T3 cell migration at the scratched area at (**a**) 4 h, (**b**) 12 h, (**c**) 24 h, and (**d**) 35 h of incubation.

**Figure 9:**
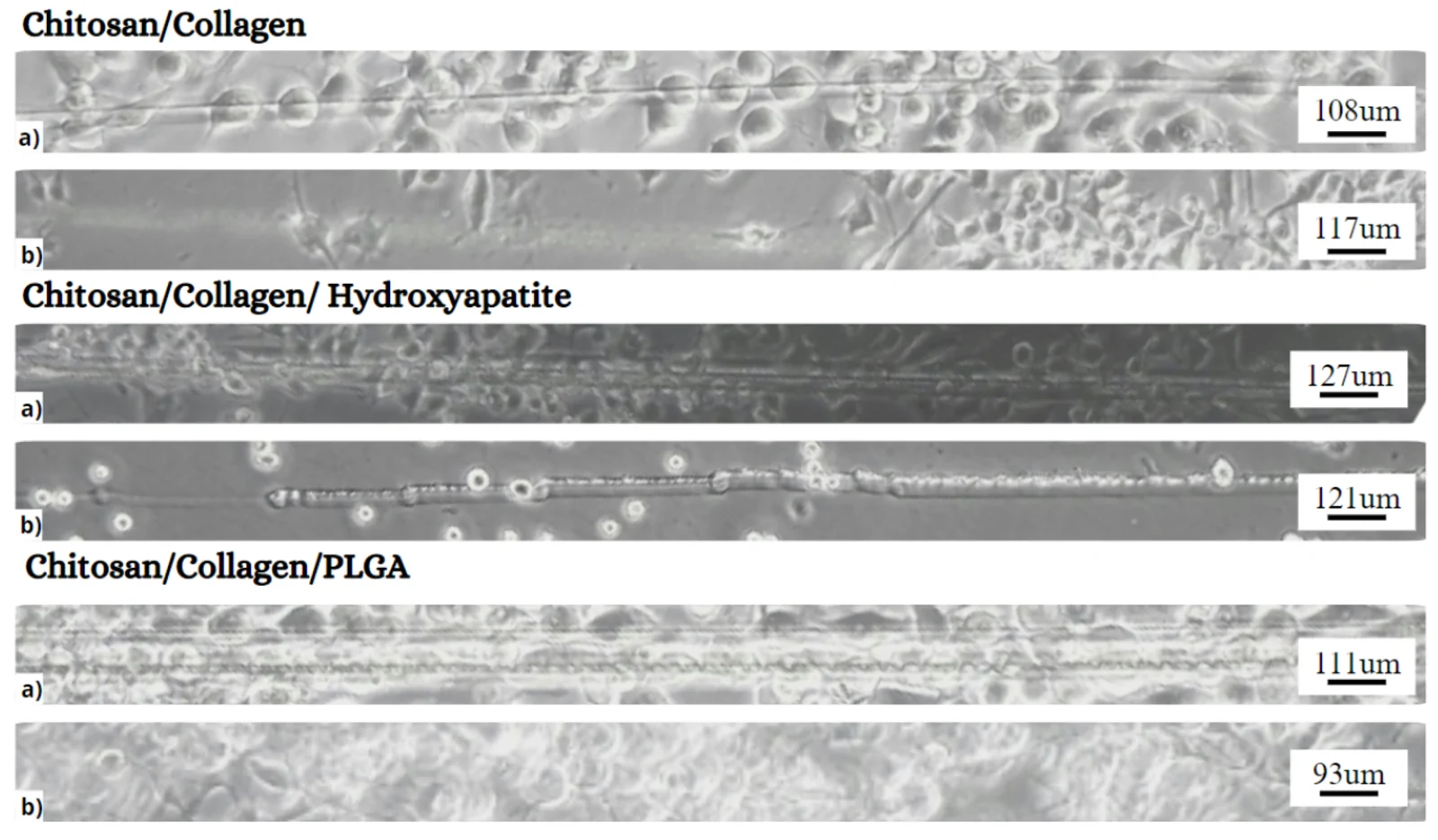
Microscopic images of the scratch assay performed with 3T3 fibroblast cells cultured on different 3D scaffold formulations: Chitosan/Collagen, Chitosan/Collagen/Hydroxyapatite, and Chitosan/Collagen/PLGA.

In the field of tissue engineering and cell migration, biomaterial scaffolds play a pivotal role in supporting the adhesion and proliferation of cells, “Cell migration assay”. This assay mimics In-vivo cell migration in mouse embryo 3T3 fibroblasts. An area is scratched to remove the cell; the resulting empty area is referred to as the “artificial wound” (See Figure 7, Figure 8 and Figure 9). This area is controlled by cell migration and the rate at which it refills. The initial adhesion of cells onto the potential scaffold (fibers) is a critical step that influences subsequent cellular activities, including migration, proliferation, and differentiation. The scratch assay used in this study quantified the number of cells observed within a predefined region of the artificial wound after 35 h. Because the NIH/3T3 cells were not treated with an antiproliferative agent and no independent proliferation assay was performed, repopulation of the scratched area reflects a combination of cell migration and proliferation.

The successful adhesion and initial growth of cells observed in Figure 7 over the 24-h period post-seeding on the Chitosan/Collagen fiber scaffolds indicate the suitability of the scaffold’s surface chemistry and topography for cellular proliferation and migration. The positive cell-material interactions suggest that the microfibrous structure provides a supportive matrix for cell attachment.

As shown in Figure 7, the 3T3 cells are represented by green circles. Continuous horizontal lines or “bands” are very representative of the collagen fibers; striation bands are characteristic of type I collagen fibers, the most common in the human body. These bands are indicative of the periodic structure of collagen, which is essential for its biological function [38].

Five culture plates were prepared for each scaffold formulation. For quantitative evaluation, the same predefined rectangular region of interest within the scratched area was applied to all scaffold-containing samples and their respective controls. Cell counts were obtained from ten images or count observations per condition at 35 h. Counting was performed manually and independently using ImageJ, 1.54t for statistical analysis. A specific area was selected to quantify cells in the wound. Results are compared with the positive and negative controls (live and dead cells). All fiber samples exhibit significant cell migration to the wound area.

After the “artificial cut” was made with a needle, the cells’ behavior was monitored for migration and proliferation in the wound area at 4, 12, 24, and 35 h.

At 0 h, no cells are present in the scratched (“wound”) area; later, at 4 h, cells exhibit limited migration, and proliferation has commenced (See Figure 8). After 24 h, there is a significant increase in cell density and migration, indicating that the fibers provide a suitable substrate for cell adhesion and proliferation. After 35 h, the cells nearly cover the wound area, showing high density and confluence on the fibers, suggesting that these materials effectively support cell growth and fibroblast migration, associated with natural wound-closure processes.

This progressive improvement in cell coverage over time underscores the potential of chitosan and collagen fibers in biomedical applications [39]. The hydrophilic nature of these polymers enables them to retain substantial moisture, which promotes cell adhesion and proliferation, as observed in the assay [40].

Qualitatively, it can be observed how the cells approach the wound area and agglomerate; all the samples exhibited migration, compared to (a) at 35 h, and (b) the control.

A one-way ANOVA including all fiber and control groups revealed a strong overall effect on cell migration (F(5,54) = 980.27, *p* < 0.0001). Tukey’s post hoc test indicated that, within each treatment, fiber-containing groups (Ch/Coll, CH/Coll/HAp, CH/Coll/PLGA) exhibited significantly higher numbers of migrated cells compared to their respective controls (all *p* < 0.001). Among the fiber types, CH/Coll/HAp (M ≈ 124) promoted the most significant migration, followed by CH/Coll/PLGA (M ≈ 117) and CH/Coll (M ≈ 72), with all pairwise differences statistically significant. Control groups remained consistently low in CH/Coll and HAp conditions (M ≈ 56). In contrast, the PLGA control presented higher values (M ≈ 153), underscoring the importance of comparing each fiber group with its matched control. These findings confirm that fiber scaffolds, especially those incorporating hydroxyapatite, enhance cellular migration into the wound area.

Another ANOVA was performed to assess significant differences among the various experimental cell groups and controls. A one-way analysis of variance (ANOVA) was performed. One-way ANOVA revealed a significant effect of fiber type on cell migration (F(2,27) = 2043.46, *p* < 0.0001, n^2^ ≈ 0.99). Tukey’s post hoc test showed that both CH/Coll/HAp (M = 124) and Ch/Coll/PLGA (M = 116) exhibited significantly higher migration compared to Ch/Coll (M = 71, *p* < 0.001). Moreover, CH/Coll/HAp demonstrated a significant increase compared to CH/Coll/PLGA (*p* < 0.001). This implies significant differences in cell counts between the treatments and their respective controls.

Specifically, treatments with CH/Coll/HAp and CH/Coll/PLGA indicate that fibroblasts cultured on Chitosan/Collagen/HAp/PLGA scaffolds exhibit higher migration rates and greater directional persistence than those cultured on conventional two-dimensional substrates. There have been reports [21] that the scaffold’s fiber structure not only mimics the native ECM architecture but also provides physical cues that promote haptotaxis and durotaxis, thereby enhancing cell motility.

The ability of Chitosan/Collagen/PLGA/HAp electrospun fibers to promote fibroblast migration is rooted in both their physical structure and biochemical composition [41]. The fibrous structure provides a physical substrate that mimics the ECM, allowing cells to adhere to the scaffold and reorganize their cytoskeleton in response to topographical cues [42]. Collagen fibers, specifically, interact with cell surface integrins and discoidin domain receptors (DDRs) to activate intracellular signaling pathways, such as the MAPK/ERK cascade, which may regulate cell proliferation and migration [43]. In parallel, chitosan’s positive charge has been reported to enhance cell adhesion through electrostatic interactions, thereby facilitating initial cell attachment and subsequent migration [44,45]. PLGA, on the other hand, serves as a carrier for potential therapeutic agents, helping ensure that the scaffold maintains its structural integrity throughout tissue regeneration. The controlled degradation of PLGA enables newly forming tissue, promoting further cell migration and ECM synthesis [46,47].

Hydroxyapatite nanoparticles were incorporated not only as an inorganic reinforcing phase but also as a bioactive calcium phosphate component that modulates cell-material interactions [48]. Hydroxyapatite surfaces can adsorb serum and extracellular-matrix-related proteins, which may promote subsequent cell adhesion and spreading; this effect is strongly dependent on the surface chemistry, charge, roughness, and topography of calcium phosphate materials. In composite scaffolds, hydroxyapatite may also increase local surface heterogeneity and provide nanoscale topographical cues that influence adhesion and migration, consistent with reports showing that scaffold topography and roughness can modulate cellular attachment and motility [49]. In our system, the higher migration observed for Chitosan/Collagen/HAp compared with Chitosan/Collagen and Chitosan/Collagen/PLGA is therefore interpreted as a literature-supported [50] consequence of the combined effects of the bioactive HAp surface, ionic interactions with the Chitosan/Collagen matrix, and altered scaffold topography. HAp-containing scaffolds produced the strongest migration response among the tested formulations, with Ch/Coll/HAp outperforming both CH/Coll and CH/Coll/PLGA systems in the in vitro scratch assay.

The combined additive of chitosan, collagen, hydroxyapatite (HAp) nanoparticles, and poly(lactic-co-glycolic acid) (PLGA) in electrospun fibers was specifically designed to address the limitations of each component [6]. For example, while collagen on its own is susceptible to rapid degradation, its combination with chitosan, HAp, and PLGA significantly enhances stability and functional performance for cell migration [51]. Furthermore, these composite fibers form a three-dimensional (3D) scaffold that facilitates improved fibroblast migration. In vitro studies using NIH 3T3 fibroblasts have demonstrated enhanced cell attachment, proliferation, and migration on Chitosan/Collagen/PLGA /HAp scaffolds compared to control substrates. Further studies should be conducted, including cytocompatibility, mechanistic analysis, and In vivo validation, to establish their suitability for tissue-engineering applications. Electrospinning is a scalable process, but clinical translation requires control of residual solvent, sterilization effects, batch reproducibility, mechanical integrity, degradation, cytotoxicity, and in vivo safety.

## 4. Conclusions

In this work, 3D scaffolds composed of Chitosan/Collagen/Poly(Lactic-Co-Glycolic Acid) (PLGA)/Hydroxyapatite (HAp) nanoparticles, fabricated via electrospinning, were used to promote cell proliferation and migration in an In vitro assay using NIH 3T3 fibroblasts from mouse embryos. Preliminary results demonstrate that the fibers provide a suitable substrate for cell adhesion and proliferation. After 35 h, the cells have nearly covered the artificial wound area, forming a high-density, confluent layer, indicating that these fibers effectively support cell growth and migration, thereby mimicking the natural wound-healing process. The fiber scaffold leverages the unique bioactive properties of chitosan and collagen, the structural stability conferred by hydroxyapatite nanoparticles, and the controlled degradability of PLGA to create an environment that supports cell adhesion, proliferation, and directed migration for biomedical applications. Our results demonstrate that the additive integration of organic–inorganic–synthetic components enhances fibroblast migration in an In vitro scratch assay, with Chitosan/Collagen/HAp scaffolds outperforming both Chitosan/Collagen and Chitosan/Collagen/PLGA systems.

## Figures and Tables

**Table 1:** Optimized parameters for the formation of fibers based on Chitosan-Collagen polymers at 120 mm of distance from the electrospinning equipment tip to the collector. Chitosan concentration (LMW CH % *w*/*v*), solvents (TFA:DCM % *v*/*v*), principal polymers (CH:Coll-${\mathrm{Sol}}_{\mathrm{cc}}$), hydroxypatite and poly(Lactic-co-Glycolic Acid concentrations (HAp:${\mathrm{Sol}}_{\mathrm{cc}}$),PLGA:${\mathrm{Sol}}_{\mathrm{cc}}$)), needle diameter, and voltage.

Formulation	CH Conc. (% *w*/*v*)	TFA:DCM Ratio	CH:Coll Ratio	HAp:Blend Ratio	PLGA:Blend Ratio	Needle Diameter (mm)	Voltage (kV)	Distance (mm)
CH	7	1:0	1:0	0	0	0.90	14–19	120
Collagen	–	1:0	0:1	0	0	0.55	11–14	120
CH/Coll	10	1:0	1:1	0	0	0.90–1.20	18	120
CH/Coll/HAp	10	1:0	1:1	1:19	0	0.55–0.70	13–14	120
CH/Coll/PLGA	10	1:0	1:1	0	1:19	0.70	14–17	120

Notes: CH, chitosan; Coll, collagen; HAp, hydroxyapatite; PLGA, poly(lactic-co-glycolic acid); TFA, trifluoroacetic acid; DCM, dichloromethane. CH concentration is expressed as % *w*/*v*. The CH:Coll, HAp:blend, and PLGA:blend columns indicate the formulation ratios used during solution preparation.

**Table 2:** Thermal degradation stages and mass losses of the electrospun scaffolds.

Thermal Event	Weight Loss (%) and Temperature Region		
	CH/Coll	CH/Coll/HAp	CH/Coll/PLGA
Moisture and residual volatiles	1.11 100 °C	5.85 low-temperature region	6.24 100 °C
Initial organic degradation	7.67 first degradation event	24.66 intermediate-temperature region	26.12 100–300 °C
Main organic-matrix degradation	25.33 DTG maximum near 250 °C	–	30.81 300–500 °C
High-temperature degradation	25.55 and 25.21 DTG maximum near 400 °C	–	29.83 500–700 °C

*Notes:* CH, chitosan; Coll, collagen; HAp, hydroxyapatite; PLGA, poly(lactic-co-glycolic acid); DTG, derivative thermogravimetry.

## Data Availability

The data that support the findings of this study are available upon reasonable request from the authors.
